# Analysis of Adverse Events and Intravenous Iron Infusion Formulations in Adults With and Without Prior Infusion Reactions

**DOI:** 10.1001/jamanetworkopen.2022.4488

**Published:** 2022-03-30

**Authors:** Asad H. Arastu, Benjamin K. Elstrott, Kylee L. Martens, Jonathan L. Cohen, Michael H. Oakes, Zhoe T. Rub, Joseph J. Aslan, Thomas G. DeLoughery, Joseph Shatzel

**Affiliations:** 1 Department of Medicine, Oregon Health & Science University, Portland; 2 Department of Hematology-Oncology, Knight Cancer Institute, Oregon Health & Science University, Portland; 3 Department of Pharmacy, Oregon Health & Science University, Portland; 4 Department of Medicine, Chicago Medical School at Rosalind Franklin University of Medicine and Science, Chicago, Illinois; 5 Department of Biomedical Engineering, Oregon Health & Science University, Portland

## Abstract

**Importance:**

Although iron deficiency is common, it remains unclear which iron repletion strategy is associated with the lowest rate of infusion-related adverse events, and how patients with history of infusion reaction should be managed.

**Objective:**

To evaluate rates of infusion reactions among 4 commonly used intravenous iron repletion strategies and determine how readministration was managed in patients with history of reaction.

**Design, Setting, and Participants:**

This cohort study included all patients receiving intravenous iron infusion from January 1, 2015, to September 7, 2021, at 6 centers in Portland, Oregon. Participants included a total of 12 237 patients with iron deficiency, not restricted by etiology. Statistical analysis was performed from September to October 2021.

**Exposures:**

Type of intravenous iron formulation and concurrent administration of diphenhydramine, epinephrine, famotidine, and/or hydrocortisone, used as surrogate maker of infusion reaction.

**Main Outcomes and Measures:**

Incidence of adverse events, including severe events requiring epinephrine, stratified by type of iron formulation, and in patients who received premedication or with history of infusion-related reaction receiving subsequent doses.

**Results:**

Among 35 737 unique iron infusions (12 237 patients [9480 (77.5%) women; 717 (5.9%) Black; 10 250 (83.7%) White; mean (SD) age of 51 (20) years]), comprising 22 309 iron sucrose doses, 9067 iron dextran total doses (1771 preceded by test dose, 56 test doses alone), 3147 ferumoxytol doses, and 1214 ferric carboxymaltose doses, incidence of adverse events was 3.9% (n = 1389; 95% CI, 3.7%-4.1%). Rate of infusion events differed among iron formulations: 4.3% (n = 970; 95% CI, 4.1%-4.6%) iron sucrose, 3.8% (n = 345, 95% CI: 3.4%-4.2%) iron dextran (test and full doses or test dose alone), 1.8% (n = 57; 95% CI, 1.4%-2.3%) ferumoxytol, and 1.4% (n = 17, 95% CI, 0.8%-2.3%) ferric carboxymaltose (*P* < .001). Severe adverse events were exceedingly rare with only 2 documented epinephrine administrations, both associated with iron dextran. Incidence of adverse events among those who received premedication was 23-fold higher compared with those who did not (38.6% vs 1.7%, χ^2^_1_ = 7324.8; *P* < .001). Among 873 patients with history of infusion reaction who underwent readministration, the majority received the same formulation, which was associated with significantly higher reaction rate particularly if premedication was administered (68% [95% CI, 64%-72%] vs 32% [95% CI, 26%-41%], respectively), compared with those who received an alternate formulation (21% [95% CI, 11%-35%] vs 5% [95% CI, 2%-12%], respectively) (*P* < .001).

**Conclusions and Relevance:**

These data, and the preponderance of published evidence, suggest that intravenous iron is generally well tolerated with exceedingly low risk of severe reaction, use of premedication and test doses are unnecessary, and that optimal prevention and management of infusion-related reactions warrant further study.

## Key Points

**Question:**

What are the overall incidence and comparative rates of infusion reactions among commonly used intravenous iron formulations?

**Findings:**

In this multicenter cohort study of 35 737 iron infusions administered to 12 237 patients, the incidence of infusion reactions was 3.9%, with only 2 total documented epinephrine administrations. There were significantly different comparative rates of infusion-related adverse events among individual iron formulations, and the use of premedication was associated with greater risk of adverse events.

**Meaning:**

These results suggest that intravenous iron administration is generally well tolerated, although it may differ among available iron formulations, with an exceedingly low risk of severe infusion reaction.

## Teaser

This cohort study evaluates rates of infusion reactions among 4 commonly used intravenous iron repletion strategies and how readministration was managed in patients with history of reaction.

## Introduction

Intravenous (IV) iron is more effective and faster acting than oral iron; however, concerns persist among some clinicians regarding the overall safety of certain IV iron formulations.^1,2,3,4^ IV iron carries a minimal (1:100-250) risk of inducing a minor hypersensitivity reaction that can include flushing, urticaria, pruritus, or chest and/or back pressure.^5,6,7,8^ Severe adverse events (SAEs) are exceedingly rare, but the exact frequency is unclear. Some have estimated the rate of anaphylaxis with IV iron to be less than 1 per 250 000 administrations.^9^ Proposed mechanisms of IV iron-associated adverse events (AEs) include type I hypersensitivity reactions to dextrans, which can be used as plasma expanders, complement activated-related pseudo-allergy (CARPA), or other non-IgE mediated mechanisms of complement activation.^10,11^

There are several commonly used formulations of IV iron, including iron sucrose (IS), low molecular weight iron dextran (LMWID), ferumoxytol, ferric gluconate, and ferric carboxymaltose, as well as the recently approved ferric derisomaltose, although the comparative rate of reactions among available formulations has not been well described and warrants further investigation.^12^ Likewise, evidence is lacking for the clinical utility of test doses of LMWID or the use of premedication to prevent infusion reactions, and few data are available to guide the management of patients who experience a reaction and require ongoing iron repletion. This ambiguity, as well as general discomfort with the use of newer infusion formulations, may lead to less patient-centric and cost-effective iron repletion strategies, such as those requiring multiple visits for low-dose iron infusions as opposed to single-dose total infusions.^13,14^

To address this ambiguity, we used infusion data from 6 centers in Portland, Oregon. First, we collected the concomitant administration of reaction medications (diphenhydramine, epinephrine, famotidine, and/or hydrocortisone) post-IV iron to gauge the rate of all iron-related AEs. We next evaluated the utility of premedication and test dosing with certain iron formulation. Lastly, we evaluated practice patterns and outcomes in patients with prior history of infusion reaction who required ongoing iron repletion.

## Methods

### Study Design and Patient Selection

We performed a multicenter cohort study of all adult patients (age ≥18 years) who received at least 1 infusion of a preselected IV iron formulation (IS, LMWID, ferumoxytol, and ferric carboxymaltose), at 6 centers in Portland, Oregon (Oregon Health & Science University [OHSU] and 5 affiliated community hematology practices in Beaverton, East Portland, Gresham, Northwest Portland, and Tualatin, Oregon) from January 1, 2015, through September 7, 2021. This study was approved by the OHSU institutional review board prior to initiation, and a waiver of informed consent was granted owing to the retrospective nature of the study. The clinical indication of IV iron administration was determined by the individual treating physician’s discretion and was not restricted in this analysis. This study followed the Strengthening the Reporting of Observational Studies in Epidemiology (STROBE) reporting guideline.

Patients were selected through a review of pharmacy administration records of IV iron (including IS, LMWID, ferumoxytol, and ferric carboxymaltose), where each IV iron infusion was defined as either 1 total dose or test dose infusion. Test doses which were administered alone were considered unique infusions, however test dose combined with total dose infusion were considered as 1 combined infusion event. Each infusion was defined as a continuous event from start of IV iron infusion through completion, followed by any subsequent monitoring for AEs. To identify patients who experienced any type of infusion-related AE, we collected the peri-infusion medication administration of diphenhydramine, epinephrine, famotidine, and hydrocortisone as a surrogate marker of reaction response. Administration times of iron and peri-infusion medications were also captured, but only medications that were administered post–iron infusion were considered to represent a true adverse reaction. A comprehensive review of the relevant electronic health record (EHR) was additionally conducted for all patients who received epinephrine to verify accuracy, characterize infusion-related symptoms, and determine the clinical outcome after epinephrine administration. No patients were required to return for follow-up (thus none were lost to follow-up), and all patients who started IV iron infusion were captured in the analysis.

After identifying the cohort of patients who received IV iron from pharmacy administration records, baseline demographics and documented history of allergies to medications, food, or contact allergy were obtained from the EHR. The type of IV iron formulation, dose, and the use of test dose LMWID were recorded, as well as type and dose of peri-infusion medication. Test dosing was not offered for other iron formulations at the included institutions. In patients who experienced AEs who went on to receive further doses of IV iron, iron formulation, and the use of premedication were additionally collected. The indication for IV iron, the setting of administration (inpatient vs outpatient), and the characterization of infusion-related symptoms triggering the administration of peri-infusion medication were unable to be obtained.

### Study Definitions and Outcomes

The primary outcome of interest in this analysis was the overall incidence of AE and SAEs among all patients who received at least 1 dose of IV iron. An AE was defined as the administration of diphenhydramine, epinephrine, famotidine, and/or hydrocortisone within 24 hours post–iron infusion. SAE was further characterized as the use of epinephrine within 24 hours of IV iron administration. Secondary outcomes included the incidence of AEs in those who received premedication, defined as the use of diphenhydramine, famotidine, and/or hydrocortisone within 24 hours preceding infusion, and in those who received test doses, defined as 25-mg IV LMWID prior to total dose LMWID infusion. Additional subgroup analyses were performed to measure the incidence of AEs in patients with documented history of allergy, as well as in patients with history of infusion-related reaction who received subsequent doses of IV iron, stratified by iron formulation and use of premedication.

### Statistical Analysis

Descriptive statistics were used to summarize baseline and outcome characteristics, with categorical variables presented as number and percentage and numerical variables presented as mean and SD. After accounting for the number of premedications and postmedications administered, Pearson χ^2^ tests were performed to determine the statistical significance of infusion reactions based on type of iron formulation. Statistical significance for analyses was set at *P* < .05, and all testing was 2-tailed. Additional Pearson χ^2^ tests were performed to compare the association of the following variables with rates of AEs: premedication, prior documented allergy, test-doses of LMWID, or readministration of IV iron after reaction. All analyses and data visualizations were performed using R for macOS version 4.1.2 (R Project for Statistical Computing) from September to October 2021.

## Results

### Baseline Patient and Infusion-Related Characteristics

A total of 35 737 unique IV iron infusions were administered to 12 237 patients during the 6.5-year study period. Baseline patient characteristics and demographic information are reported in Table 1. Among 12 237 patients included in the analysis, 9480 (77.5%) were women; 717 (5.9%) were Black, 10 250 (83.7%) were White; the mean (SD) age was 51 (20) years; and 9677 (79.1%) had a documented history of at least 1 allergy (medication, food, or contact allergy) in the EHR. Among IV iron formulations administered, a total of 22 309 infusions (62.4%) were IS, 9011 (25.2%) were LMWID (of which 1715 [19.0%] were preceded by a test dose), 56 (0.16%) were test dose LMWID alone, 3147 (8.8%) were ferumoxytol, and 1214 (3.4%) were ferric carboxymaltose.

**Table 1.** Patient Demographics, Clinical Information, and Infusion Events

Characteristic	Patients, No. (%) (N = 12 237 )
Women	9480 (77.5)
Men	2757 (22.5)
Age at time of infusion, mean (SD), y	51 (20)
Documented history of allergy^a^	9677 (79.1)
Race	
American Indian/Alaska Native	119 (1.0)
Asian	338 (2.8)
Black	717 (5.9)
White	10 250 (83.7)
Unknown, declined, or other^b^	813 (6.6)
Ethnicity	
Hispanic	1026 (8.4)
Non-Hispanic	10 161 (83.0)
Unknown or declined	1050 (8.6)
Total iron infusion events, No.	35 737
Iron sucrose	22 309 (62.4)
Iron dextran full dose (with or without test)	9011 (25.2)
Iron dextran test alone	56 (0.2)
Ferumoxytol	3147 (8.8)
Ferric carboxymaltose	1214 (3.4)

^a^ Inclusive of allergy to medication, food, or contact allergies.

^b^ Data were not captured for specific race categories included in the other race category, which were limited to the electronic health record inputs.

### Cumulative Incidence of IV Iron-Related AE and SAEs

The cumulative incidence of infusion-related AEs was 3.9% (95% CI, 3.7%-4.1%; 1389 reactions) among 35 737 IV iron infusions included in the analysis. Notably, the rate of infusion reactions differed significantly by IV iron formulation. We detected reaction rates of 4.3% (95% CI, 4.1%-4.6%; 970 reactions) in those who received IS, 3.8% (95% CI, 3.4%-4.2%; 345 reactions) in LMWID (test and full dose or test dose alone), 1.8% (95% CI, 1.4%-2.3%; 57 reactions) in ferumoxytol, and 1.4% (95% CI, 0.8%-2.3%; 17 reactions) in ferric carboxymaltose (*P* < .001). Notably, our study was not specifically designed with intent to compare the rate of AEs between individual formulations.

Per-patient epinephrine use, indicating SAEs, was exceedingly rare with only 2 events occurring in the LMWID group and 0 with other iron formulation groups. Table 2 shows the rates of AEs and SAEs stratified by IV iron formulation. The first patient, a female aged between 40 and 50 years with no significant past medical history, experienced nasal congestion that progressed to throat swelling. She received both diphenhydramine and famotidine but continued to experience a throat swelling sensation. She subsequently received steroids and epinephrine, was transferred to the emergency department with symptom resolution and did not require further intervention or hospitalization. The second patient, a female aged between 60 and 70 years with underlying interstitial lung disease and baseline 2 L/min oxygen requirement, developed back pain, chest and throat tightness, and wheezing following IV iron infusion. She too initially received diphenhydramine, followed by famotidine, albuterol, and solumedrol. She became increasingly tachycardic (heart rates between 150 and 160 beats/min) with oxygen desaturation to 74%, requiring escalation to 4 L/min oxygen supplementation. Epinephrine was administered and she was transferred to the emergency department, symptoms resolved during evaluation, and she did not require inpatient hospitalization.

**Table 2.** Rates of Adverse Events Stratified by Intravenous Iron Formulation

Postinfusion medication use	Infusions, No./total No. (% of reactions)					*P* value
	Total	IS	LMWID^a^	Ferumoxytol	Ferric carboxymaltose	
Iron reactions^b^	1389/35 737 (3.9)	970/22 309 (4.3)	345/9067 (3.8)	57/3147 (1.8)	17/1214 (1.4)	<.001^c^
Epinephrine	2/1360 (0.1)	0	2	0	0	NA
Diphenhydramine	664/1360 (48.8)	400	217	34	13	NA
Famotidine	858/1360 (63.0)	601	217	30	10	NA
Hydrocortisone	245/1360 (18.0)	134	96	12	3	NA

Abbreviations: IS, iron sucrose; LMWID, low molecular weight iron dextran; NA, not applicable.

^a^ Includes combined test and full, full dose alone, and test dose alone.

^b^ Defined by the use of epinephrine, diphenhydramine, famotidine, and/or hydrocortisone within 24 hours postinfusion. Multiple postinfusion medications were provided for some reactions; the sum of medication uses will exceed the total number of iron reactions.

^c^ Pearson χ^2^ test.

### Secondary Outcomes of Interest

We next evaluated the incidence of AEs among those who received at least 1 premedication within 24 hours of IV iron infusion. A total of 2157 iron infusions (6.0%) were associated with the administration of at least 1 premedication (diphenhydramine, 1085 [50.3%]; famotidine, 881 [40.8%], or hydrocortisone, 600 [27.8%]). The incidence of AEs among those who received premedication was approximately 23-fold higher compared with those who did not (38.6% vs 1.7%; χ^2^_1_ = 7324.8; *P* < .001) (Table 3). Postmedication administration with subsequent doses of IV iron is shown in the Figure.

**Table 3.** Rates of Adverse Events Among Patients Receiving Premedication or Iron Dextran Test Doses

	Total doses, No.	Infusion reaction, No. (%)	No infusion reaction, No (%).	*P* value
Rates of infusion reaction by premedication status				
Premedication given	2157	833 (38.6)	1324 (61.4)	<.001^a^
No premedication given	33 580	556 (1.7)	33 024 (98.3)	
Rates of infusion reaction in iron dextran group by test-dose status				
Full dose only	7296	279 (3.8)	7017 (96.2)	.90^a^
Test and full dose intended	1771	66 (3.7)	1705 (96.3)	
Test dose only^b^	56	29 (51.8)	27 (48.2)	NA

NA, not applicable.

^a^ Pearson χ^2^ test.

^b^ Test dose–only patients are included in the test and full dose intended category above. Twenty-seven patients did not have infusion reactions but also did not go on to receive the intended full dose within 24 hours.

**Figure.**
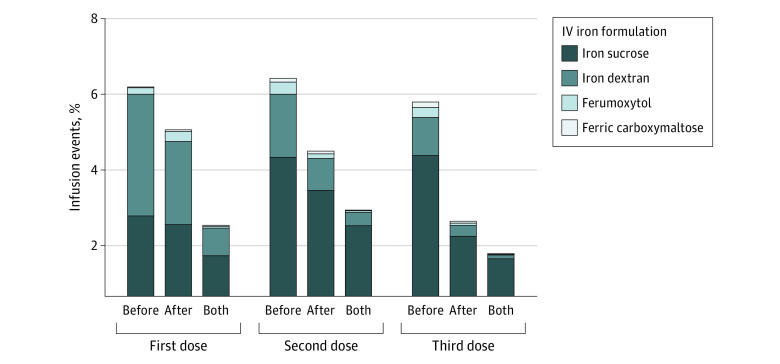
Medication Use Preinfusion, Postinfusion, or Both Given for Subsequent Intravenous (IV) Iron Doses The figure shows medication use preinfusion (before), postinfusion (after), or both given for subsequent IV iron doses.

The incidence of AEs associated with the use of test dose LMWID was also analyzed, and outcomes reported in Table 3. Among 9067 iron dextran total doses, test doses were intended to be given prior to 1771 infusions (19.5%), whereas total doses without test doses were administered in 7296 infusion events. There was no significant difference in rates of AEs between total dose alone (n = 7296 [3.8%]) and test dose followed by total dose groups (n = 1715 [3.7%]) . Within the intended test dose followed by total dose group, a total of 56 patients received only the test dose, of whom 29 (52%) received a post–test dose reaction medication.

### Additional Subgroup Analyses

We performed additional subgroup analyses using stricter cohort selection criteria, summarized in Table 4. In the first analysis, we evaluated the rate of AEs occurring among 9677 patients with documented history of allergy. Of the 25 144 infusions (70.8%) occurring in patients with history of allergy, a significantly higher rate of AEs was detected compared with those without history of allergy (4.2% [95% CI, 4.1%-4.6%] vs 3.2% [95% CI, 3.0%-3.6%], *P* < .001).

**Table 4.** Subgroup Analysis of Patients With History of Adverse Events and Rates of Repeat Infusion Reaction With Retrial of Intravenous Iron by Various Management Methods

Formulation decision	Premedication status	Repeat infusion reaction, No. (%)	No repeat infusion reaction, No. (%)	*P* value
Retrial of same formulation	Not premedicated	48 (32)	102 (68)	<.001^a^
	Premedicated	411 (68)	194 (32)	
Switch to new formulation	Not premedicated	4 (5)	75 (32)	
	Premedicated	8 (21)	31 (79)	

^a^ Pearson χ^2^ test.

To gain a greater understanding of practice variations in patients with prior history of an infusion reaction receiving ongoing iron repletion, an additional subgroup analysis of those who underwent readministration of IV iron was performed. Among 873 patients who met inclusion criteria and underwent subsequent iron administration, 755 (86%) received the same iron formulation (of whom 605 [80%] received premedication), whereas 118 (14%) were switched to an alternate formulation (of whom 39 [33%] received premedication). Rates of recurrent AEs were significantly higher in the cohort of patients who underwent retrial of the same IV formulation and were premedicated vs not (68% [95% CI, 64%-72%] vs 32% [95% CI, 26%-41%], respectively), compared with those who were switched to an alternative IV formulation and were premedicated vs not (21% [95% CI, 11%-35%] vs 5% [95% CI, 2%-12%], respectively) (*P* < .001).

## Discussion

In this multicenter cohort study of more than 12 000 patients receiving 35 737 unique iron infusions, AEs were detected in only 3.9% (n = 1389) of all infusion events. The rate of SAEs, defined by the use of epinephrine within 24 hours postinfusion, was exceedingly low with only 2 occurrences in total, both associated with IV LMWID. Although we cannot be completely certain given the retrospective nature of this review, it is possible that intervention with diphenhydramine and epinephrine converted a minor reaction into a more hemodynamically meaningful event ostensibly because of iron. There was a statistically significant difference in rate of AEs among the 4 commonly used IV iron formulations that were included in the analysis, with the highest rate of infusion reaction occurring among those who received IS (4.3%) and lowest among ferric carboxymaltose (1.4%). Among those who received total dose LMWID, the majority were administered as total dose alone with only 19.5% (n = 1771) preceded by test dose administration, and 56 infusions were given as test dose only. Notably, there was no significant difference in infusion-related AEs between total dose LMWID administration approaches (with or without test dose). The findings in our study suggest that IV iron administration is generally well tolerated with a low adverse reaction rate, and the routine use of test doses and premedications are likely of limited benefit.

Our study adds to the already robust body of evidence supporting the safety of IV iron infusion. Older formulations of IV iron, particularly the high molecular weight iron dextran (HMWID) formulation Dexferrum, which was first made available in 1996, was historically associated with unacceptably high rates of allergic and hypersensitivity reactions.^2,11,13,15^ Although not entirely elucidated, this has largely been attributed to IgE-mediated, type 1 hypersensitivity response to the dextran group leading to basophil degranulation with subsequent histamine release, as well as specific IgE and IgG antibody formation against the dextran group.^10,16,17^ This agent was subsequently removed from formulary owing to safety concerns.

With the advent of newer formulations of IV iron, infusion reactions have not only substantially declined, but alternate mechanisms driving these reactions have also been postulated. One theory suggests that the transient presence of free iron in circulation, caused by iron extrusion from the carbohydrate complex, can occur in situations when IV iron is infused too rapidly to be safely bound to transferrin.^11^ Also of note is the concept of CARPA via non-IgE mediation, where C3a and C5a bind to mast cells, basophils, and macrophages, triggering the release of cytokines responsible for the clinical features of a hypersensitivity reaction.^10,11^ Although traditionally the proposed driver of hypersensitivity reactions associated with monoclonal antibody administration, CARPA has also been associated with nanoparticle-containing drug reactions of which all available IV iron formulations now consist.^18^ Hempel et al^19^ attempted to analyze IV iron complement activation by comparing 5 IV iron formulations using functional complement assays both in vitro and ex vivo, of which both LMWID and ferric carboxymaltose demonstrated complement-activating potential. It remains unclear whether free iron itself or its carbohydrate shell is responsible for complement activation, although the known association between nanoparticle activation of complement suggests that the carbohydrate shell may in fact be the primary culprit.^11^

Risk factors that appear to both increase the incidence and severity of hypersensitivity reactions to IV iron include history of allergy or atopy, a fast infusion rate, and prior history of reaction to IV iron.^10,18^ Our study also found a significantly higher rate of infusion reaction in patients with documented history of allergies, although it should be noted that we captured a higher-than-expected rate of documented allergies, likely owing to the inclusion of allergies to medications, food, and contact allergy. Because of the proposed risk of hypersensitivity to the dextran-component and an association of LMWID with complement-activation in vitro,^19^ dextran-free formulations of IV iron had gained favor due to a theoretical lower risk of anaphylactoid reactions, although oftentimes at the expense of sacrificing single-dose total infusion for multiple visits of low-dose iron. This theory, however, has largely been debunked by retrospective and meta-analyses of patients undergoing IV iron infusion, in which LMWID has consistently demonstrated similar if not decreased rates of AEs compared with similar formulations.^20,21,22^ The context of this bias is perhaps related to the historical use of HMWID and its association with SAEs, although it is now well known that higher molecular weight compounds confer a greater degree of immunologic propensity.^23^ Furthermore, there remains a paucity of high-quality evidence supporting the use of test doses of IV LMWID, which may mark a somewhat antiquated approach to administration driven by previous biases, with more recent data demonstrating the safety of single-dose IV 1000 mg administration.^24^ In fact, our study found no significant difference in infusion reactions between those who did and did not receive a test dose, although 29 of the patients who had AEs to a test dose requiring postinfusion medications did not proceed to the full dose. Overall, the low and relatively similar rate of AEs between these strategies calls into question the overall clinical utility of the test dose approach.

Randomized clinical trials (RCTs) comparing rates of infusion reactions among all available formulations are generally lacking, and results of various retrospective analyses often demonstrate conflicting results. Our study found significant differences in the rate of infusion reactions among IV iron formulations, with the highest rates of reaction occurring among those who received IS (4.3%) and lowest among those who received ferric carboxymaltose (1.4%). A retrospective study of 619 patients who received 5 different IV iron formulations reached similar conclusions, with a significantly higher odds ratio of AE occurring in those who received IS (OR, 5.7; 95% CI, 1.6-21.3).^22^ Iron sucrose differs from other formulations in that the smaller carbohydrate core binds iron less tightly, resulting in significantly increased labile-free iron after administration and higher risk of hypersensitivity reaction.^13^ With the advent of multiple low doses of IS, most studies have demonstrated similar safety profiles when compared with LMWID.^23,25,26^ Additional studies have evaluated newer formulations of IV iron, including the FIRM study, a recent randomized, double-blinded, controlled trial of nearly 2000 enrolled patients comparing the safety of ferumoxytol and ferric carboxymaltose, which detected no significant difference in the primary end point of moderate-to-severe AEs or hypotension (0.6% vs 0.7% in ferumoxytol and ferric carboxymaltose, respectively) and no anaphylaxis reported in either group.^27^ An often cited report issued by the European Medicines Agency in 2013 suggests that although available data shows a clear association with IV iron and hypersensitivity reactions, the data cannot be used to detect differences in rates of reactions between iron formulations.^28^ Furthermore, given the very low rate of SAEs, they conclude that the benefits of IV iron administration outweigh its risks, provided that measures are taken to minimize severe events.

Similarly, the use of premedications, such as corticosteroids and antihistamines, to prevent or minimize infusion-related AEs remains highly controversial. Not only has the efficacy of premedications been questioned, but there is data to suggest that the first-generation of histamine receptor antagonists, such as diphenhydramine, account for a large majority of perceived mild hypersensitivity reactions to IV iron.^29,30^ These agents routinely cause somnolence, flushing, hypotension, and tachycardia that mimic infusion-related reactions leading to a higher frequency of reported AEs.^5^ Even second-generation antihistamines, though less sedating, have also been associated with flushing, palpitations, and dizziness. These conclusions are consistent with our study, in which premedications were administered in 6.0% (n = 2157) of all infusion events and were associated with a 23-fold higher rate of infusion reaction compared with no premedication (38.6% vs 1.7%, *P* < .001). Interestingly, similar findings were seen when we evaluated the subset of patients with history of infusion reaction who received ongoing iron repletion. AEs were significantly greater in patients who received the same IV iron formulation compared with those who switched to an alternate formulation, although notably higher rates of AEs in both groups were detected in those who were premedicated, supporting the theory that many of the documented reactions may instead be because of premedication itself, and not iron. Adding to a growing body of data to support the safety of IV iron rechallenge following an AE is a recently published large, retrospective cohort analysis conducted in Australia by Stojanovic et al.^31^ Sixty-nine patients who experienced an AE to IV iron polymaltose who did not complete their initial infusion were subsequently rechallenged with ferric carboxymaltose, of which 68 patients tolerated completion without incident, including 3 patients who experienced SAEs during their first IV iron infusion. General conclusions from these and other studies suggest that routine use of premedications be limited only to those with substantial risk factors (such as previous reaction to IV iron, fast infusion rate, multiple drug allergies, severe asthma or atopy^10^), rechallenge to an alternate IV iron formulation is safe, and ultimately, future RCTs are needed to adequately study the ideal approach to both prevention and management of infusion-related reactions among all currently available IV iron formulations.

Our study has several strengths. First, to our knowledge, this is the largest retrospective analysis comparing IV iron infusion-related reactions among 4 commonly used formulations. Second, we believe in the methods of our data accrual approach in detecting clinically relevant infusion-related reactions through the identification of perimedication administration. Furthermore, rates of adverse events in our study are consistent with prior estimates, instilling confidence that our findings are reproducible when compared with previously published data.

### Limitations

This study had some limitations. There are limitations inherent to a retrospective analysis and data extraction, which include the inability to randomize patients by IV iron formulation and capture patient- and infusion-specific details, including type of clinical reaction to identified allergens and infusion rates of each IV iron administration. Second, we acknowledge that the methods used in this analysis cannot fully account for confounding by indication, which can lead to biased outcome estimates in retrospective studies and is of particular concern when comparing infusion reaction rates with the use of premedications, which has independently been associated with higher rates of AEs in those receiving IV iron. However, this concern is mitigated by a subgroup analysis of patients with prior reaction to IV iron who underwent readministration, in which we reached similar conclusions. Additionally, we are limited by the use of postmedication administration as a surrogate marker of AEs and were unable to verify the accuracy of these findings with individual chart review given the large sample size. However, we were able to undertake comprehensive chart review to verify the accuracy of SAEs in patients who received epinephrine, which may in fact have not been clinically indicated and potentially converted a minor reaction into a more hemodynamically significant event. Finally, although the higher rates of AEs detected among those who received premedication may be attributed to symptoms that mimic infusion-related reaction, it is also possible that we inadvertently captured some medications that were administered during hospitalization. This concern is tempered by the fact that the vast majority of IV iron is given during ambulatory encounters at our relevant institutions, and as such the included peri-infusion medications evaluated in our study are highly likely to be IV iron–related administrations. Based on our practice, we anticipate a very low rate of inpatient IV iron administration (likely less than a few percent), although unfortunately we were not able to measure this rate given limitations of retrospective data collection through the EHR.

## Conclusions

This cohort study found that patients receiving IV iron had very low rates of infusion-related reactions, with a near-zero rate of epinephrine administration. The routine use of premedications, particularly first-generation histamine receptor antagonists, may in fact cause more harm than benefit by inducing symptoms that could be perceived as a mild hypersensitivity reaction. Although further investigation is warranted to determine the ideal approach of readministration of iron in those who experience an infusion reaction, our data suggests that rechallenging patients with IV iron, perhaps with an alternate formulation and not preceded by sedating antihistamines, would be both safe and effective. Overall, IV iron continues to hold an undeniably essential role in the management of iron deficiency.
