# Error in Byline

**DOI:** 10.1001/jamanetworkopen.2022.21567

**Published:** 2022-06-22

In the Original Investigation titled “Analysis of Adverse Events and Intravenous Iron Infusion Formulations in Adults With and Without Prior Infusion Reactions,”^1^ published March 30, 2022, a co-author’s name was misspelled. The name should have been spelled as Joseph E. Aslan, PhD. This article has been corrected.^1^
